# Creosote Bush (*Larrea tridentata*) Extract Assessment as a Green Antioxidant for Biodiesel

**DOI:** 10.3390/molecules24091786

**Published:** 2019-05-08

**Authors:** Carlos A. Sagaste, Gisela Montero, Marcos A. Coronado, José R. Ayala, José Á. León, Conrado García, Benjamín A. Rojano, Stephania Rosales, Daniela G. Montes

**Affiliations:** 1 Universidad Autónoma de Baja California, Instituto de Ingeniería, Blvd. Benito Juárez, Insurgentes Este, 21280 Mexicali, Mexico; carlos.sagaste@uabc.edu.mx (C.A.S.); marcos.coronado@uabc.edu.mx (M.A.C.); ramon.ayala@uabc.edu.mx (J.R.A.); jose.leon30@uabc.edu.mx (J.Á.L.); cnrdgarciag@uabc.edu.mx (C.G.); dmontes35@uabc.edu.mx (D.G.M.); 2 Universidad Nacional de Colombia-Medellín, Facultad de Ciencias, Laboratorio Ciencias de los Alimentos, Calle 59 no. 63-20, 050034 Medellín, Colombia; brojano@unal.edu.co (B.A.R.); srosalesd@unal.edu.co (S.R.)

**Keywords:** biodiesel, oxidative stability, *Larrea tridentata*, green antioxidant, green chemistry, biofuels

## Abstract

In this work, the antioxidant properties of methanolic extract of *Larrea tridentata* were assessed through the free radical scavenging method, ferric reducing antioxidant power and oxygen radical absorbance capacity. The phenolic acids content in the extract was quantified by high-performance liquid chromatography (HPLC) and the total phenol content by the Folin–Ciocalteu method. The extract was used as an antioxidant in biodiesel from canola oil composed mostly by fatty acid methyl esters identified and quantified by gas chromatography-mass spectrophotometry (GC-MS). The performance of the extract as an antioxidant was assessed by the oxidative stability index (OSI) with a Rancimat equipment at 100, 110, 120 and 130 °C. Additionally, the change of the peroxide value (PV) and the higher heating value under conditions of oxidative stress at 100 °C and air injection were measured. The antioxidant capacity of the extract reached 50,000 TAEC (micromole of Trolox antioxidant equivalent capacity per gram). The biodiesel was constituted by more than 70% of unsaturated fatty acid methyl esters (FAME), mainly methyl oleate. The time needed to reach a PV of 100 meqO_2_/kg was almost four times longer with an antioxidant concentration of 250 mg/L than the blank. The biodiesel showed an OSI time of 1.25 h at 110 °C, while it increased to 8.8, 15.89 and 32.27 h with the antioxidant at concentrations of 250, 500 and 1000 mg/L, respectively. The methanolic *Larrea tridentata* extract proved to have an antioxidant capacity and it is a green antioxidant in biodiesel to increase its oxidative stability. According to the results obtained, the *L. tridentata* methanolic extract is an alternative to the commercial synthetic antioxidants used in biodiesel nowadays.

## 1. Introduction

In the search for alternatives to fossil fuels, humankind has chanced upon different as well as profitable and efficient options. This search is composed of economic, environmental and technical parts; however, the most urgent one is the immediate necessity of reducing the emission of greenhouse gases. Biofuels have been some of the most utilized alternatives to oil by-products.

Biofuels can be found in solid, liquid or gas form. Biodiesel is one of the most used liquid biofuels. It is a fuel composed of fatty acid alkyl esters mix, typically methyl (FAME) or ethyl esters. It is obtained from transesterification of fatty acids with short-chain alcohols in the presence of a catalyst, (usually an alkaline), with glycerol as a by-product.

Biodiesel is obtained from different materials such as vegetable oils or animal fats, e.g., canola oil, castor oil, jatropha, soybean, waste vegetable oil, lard, tallow, yellow grease, among others. The chemical composition of the biodiesel varies according to the feedstock used for its production. Biodiesel is produced often with non-edible raw oils because it does not compete with the food production, thereby it can be considered a non-toxic, sustainable and eco-friendly alternative to diesel. However, these beneficial qualities can also be a disadvantage for biodiesel itself. Besides its incompatibility with certain materials [1,2,3,4], the natural origin of the raw materials (fats and oils) used to produce biodiesel, it makes the biofuel more susceptible to deterioration by microorganisms, light and oxidation than diesel [5,6]. Thus, it is necessary to improve the biodiesel properties to increase its shelf life together with its competitiveness.

**Figure 1:**
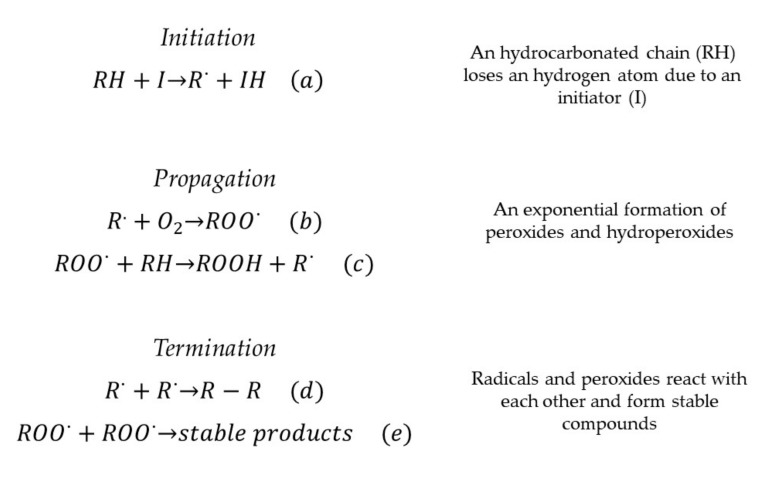
Oxidation process of biodiesel.

The process of biodiesel oxidation has been studied in the past. The process is undertaken in three phases, shown in Figure 1. In the initiation phase (Figure 1a), carbonyl radicals are made after a proton in the alkyl chain of biodiesel is removed by an initiator ion, a metallic catalyst, thermal stress or light. Generally, chains with one or more unsaturated bonds can be more susceptible to this process [7,8].

The next phase is called propagation (Figure 1b,c) because, after a slow start oxidation process, it acquires an exponential behavior. In this phase, radicals formed in the initiation phase react with O_2_ to create peroxides, which tear other protons apart from other molecules to produce hydroperoxides.

The termination phase (Figure 1d,e) takes place when the radicals previously created start reacting with each other to obtain stable compounds as final products. Secondary oxidation can be performed after this stage, where those degradation products can be oxidized until they produce aldehydes, ketones and short-chain fatty acids, the ones responsible for biodiesel acidity increase. Additionally, the reactions of secondary polymerization affect the rheological properties of biodiesel. There are many analytic methods and standards for the measurement of the oxidative stability of biofuel. Some of the most noted are the Rancimat method, the thermogravimetric analysis and the measurement of different parameters that prove the production of biodiesel degradation products, among which peroxide value stands out [9,10,11].

Antioxidants from different origins have been used to face these problems [12,13]. An antioxidant is described as a substance capable of inhibiting, delaying or interrupting any of the previously mentioned reactions [14]. Nonetheless, it is important to highlight the use of green inhibitors and inhibitors from natural sources, such as caffeic acid to inhibit corrosion [15,16] or commercial rosemary extract to increase the oxidative stability of biodiesel or other oils [17]. Many of the substances present in extracts and essential oils can act as antioxidants. They have one or more aromatic rings, hydroxyl groups, electrons in resonance or ionizable protons within their molecular structure, each of them capable of interrupting the previously described reactions [18,19]. These allow the molecule of interest, in this case the biodiesel, to stay stable for a longer time.

**Figure 2:**
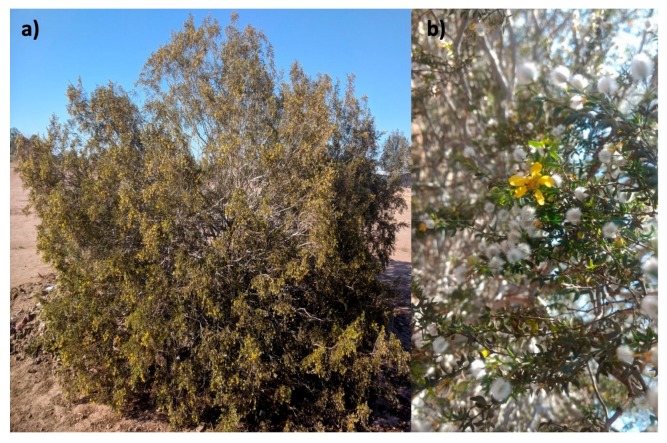
*Larrea tridentata* from Valley of Mexicali: (**a**) bush; (**b**) magnified picture of fruits, branches and flowers. (original photo).

*Larrea tridentata,* depicted in Figure 2 is a plant species whose extracts could be used as an antioxidant for biodiesel. It belongs to the Zygophyllales order and the Zygophillaceae family. It is a green bush of medium height with leaflets of 15 × 8 mm and has brightly yellow-colored solitary flowers of up to 2.5 cm in diameter that bear oval white velvety fruits. *L. tridentata* is located in the arid zones of northern Mexico and the southern U.S., at an altitude of up to 1800 m above mean sea level. Its most noted characteristics are its resistance to desiccation and intense odor, along with its ability to displace other species by aggressive competition and a nearly complete absence of natural enemies. It has been commonly named creosote bush and gobernadora in Spanish, due to its abundance in desert areas, mainly in Baja California, Mexico [20,21]. Preceding studies have proven the antimicrobial and antioxidant properties, as well as many applications in Native American medicine [22,23,24,25,26,27] of *L. tridentata*. Just as in other species from the same genus, the presence of diverse compounds such as lignans, flavonoids and terpenes has been found [28].

**Figure 3:**
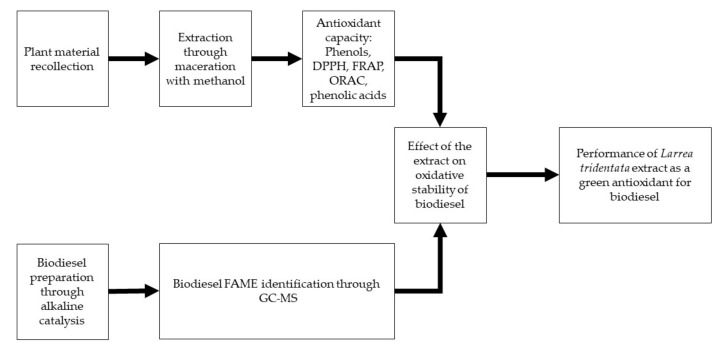
Flowchart of the methodology.

Therefore, the antioxidant properties of the methanol extract from the *L. tridentata* plant were evaluated in this work, picked up from the Valley of Mexicali, in the Colorado River Delta. The antioxidant properties of the extract were determined by the free radical scavenging method (DPPH), the ferric reducing antioxidant power (FRAP) and the oxygen radical absorbance capacity (ORAC), together with its phenol content by Folin–Ciocalteu procedure. Once it was finished, the extract was used as an antioxidant in low concentrations. The aim of this article was to assess the *L. tridentata* methanolic extract as a green antioxidant in canola biodiesel. The antioxidant effect on biodiesel oxidative stability was evaluated by peroxide value (PV) monitoring, change in higher heating value (HHV) and the oxidative stability index (OSI). The tests performed in the current work are presented in Figure 3.

## 2. Results

### 2.1. Extract Obtaining

The extract obtained was a semisolid resin, viscous enough to adhere firmly to the recipient surface. It also had a strong characteristic smell and tendency to form a foam when diluted with water or other solvents. The extraction yield was 0.7% *w*/*w*.

### 2.2. Antioxidant Properties of the Extract

The assays showed significant antioxidant activity, according to Table 1. In the ORAC assay, antioxidant activity was measured in more than 50,000 TEAC, which means that a gram of this extract is up to 12 times more efficient in protecting fluorescein than Trolox^®^. In the same way, the extract showed activity in DPPH and FRAP assays, which measured the radical scavenging and reducing capacities. The reducing capacity turned out to be 17 times higher in TEAC. The difference of results in each method can be explained according to the mechanisms intervening, both react mainly by electron transference; however, the DPPH method acts through a proton donation mechanism as well [29].

The differences in mechanisms of each method explain the differences in results. The basis of the ORAC method is to avoid oxidation of a substance, being fluorescein in this case, instead of reducing a radical, a metal or donating a proton or electron, as the other used methods.

**Figure 4:**
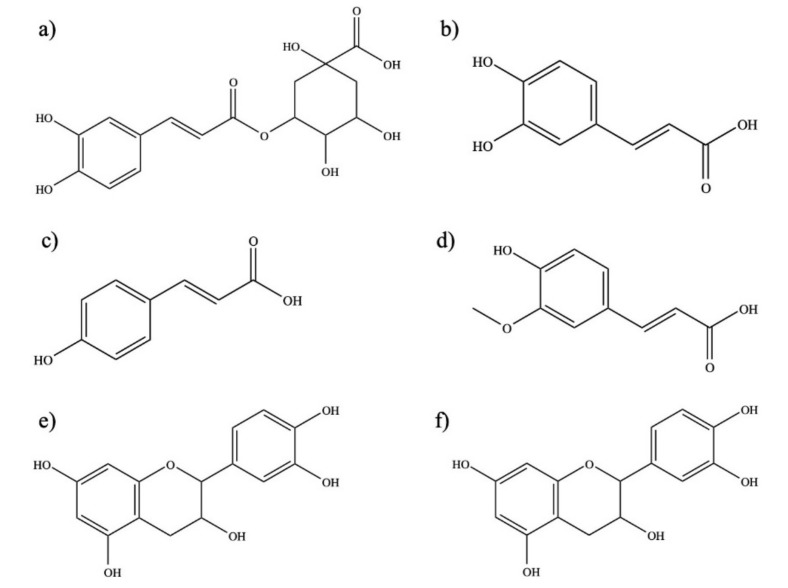
Phenolic compounds identified in methanolic *L. tridentata* extract. (**a**) Chlorogenic acid; (**b**) Caffeic acid; (**c**) *p*-coumaric acid; (**d**) Ferulic acid; (**e**) Catechin; (**f**) Epicatechin.

The extract analysis by HPLC showed the presence of phenolic acids mainly were caffeic acid. Other phenolic acids identified were p-coumaric, ferulic and chlorogenic, as well as catechin and epicatechin. The results are displayed in Table 2, and molecular structures [30] of these compounds are shown in Figure 4.

On the other side, the total phenols were 211.18 GAE. Phenolic compounds, in general, are described as the main responsible agents of antioxidant activity, yet they are not the only ones. It was asserted by Turner et al. [28], who affirmed diverse compounds could be responsible for the antioxidant activity of the extracts of *L. divaricata*. These authors proved through the DPPH method that the aqueous extract of this plant has an antioxidant capacity higher than some pure phenolic acids. Its antioxidant capacity evidences some synergistic effect of these compounds with others present in extracts. Additionally, it highlights the capacity of the extract of preventing the oxidation of the linoleic acid through the DPPH method and others.

### 2.3. Biodiesel Preparation and Characterization

The determined biodiesel composition by GC-MS displayed in Table 3, can be used to infer the original canola oil. The chemical composition of the oils varies depending on several factors: climate, geographic position and soil. Therefore, it affects the composition of biodiesel, even using the same raw material. The results showed that over 90% of biodiesel was FAME, mostly five fatty acid methyl esters: oleic, linoleic, palmitic, stearic and gondoic, in descending order. Oleic acid methyl ester had the highest proportion of biodiesel with 46%, in addition to a mix of another 16 methyl esters, summing up to 4%. It means that there are more unsaturated fatty acids, which are typically more susceptible to oxidation than saturated fatty acids. The chemical composition of biodiesel is dependent on the raw material used in its production. For that reason, these FAME are suitable to analyze oxidative stability and obtain a way to improve it, considering the equations proposed by Park et al. [7], the higher linoleic acid methyl ester concentration, the lower biodiesel stability in accelerated conditions using the Rancimat equipment. Additionally, these authors stated that oleic acid methyl ester contributes to the improvement of stability, although they did not propose any equations to measure the effect.

### 2.4. Oxidative Stability of Biodiesel

**Figure 5:**
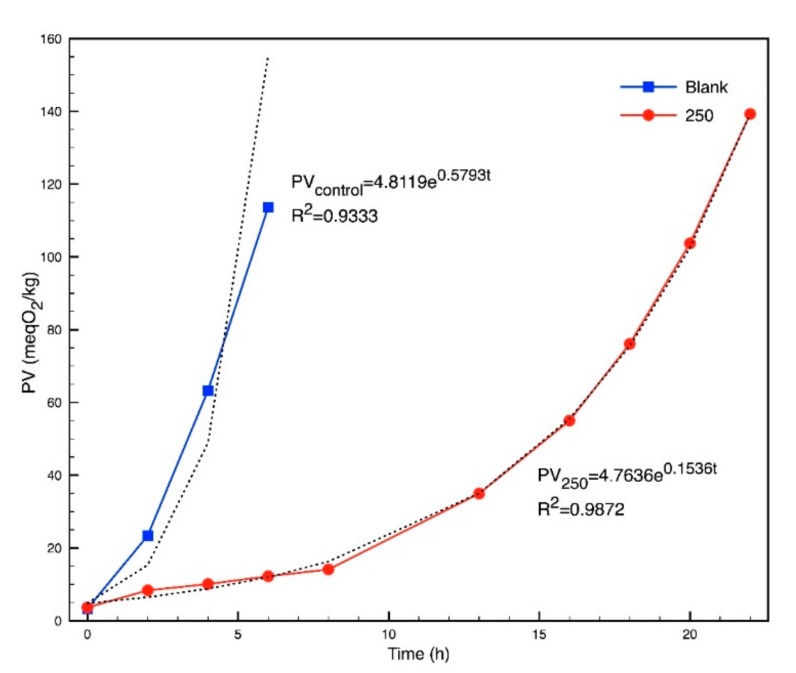
Peroxide value (PV) behavior in the active oxygen method (AOM) assay and exponential regression equations.

The AOM assay showed that biodiesel samples with over 250 mg/L of extract remained stable for more than 24 h, in conformity with PV monitoring, which did not exceed 20 meO_2_/kg. For this reason, the exponential phase of the formation of peroxide was not reached at that time. PV exceeded 100 meO_2_/kg, both in the blank solution and the 250 mg/mL concentration, taking 5.24 and 19.84 h, respectively. Results were obtained from the regression equations with high correlation coefficients, where t is the time in hours as is displayed in Figure 5.

This data was used to determine that a 250 mg/L dose can extend 3.79 times the necessary time to reach a PV of 100 meO_2_/kg, which is typical after storing biodiesel for several months [31,32]. If the present results apply to biodiesel storage at room temperature, this fuel could preserve its properties for a longer time, considering that it tends to be more stable at room temperature.

PV was also monitored by other authors who aged biodiesel at 43 °C. They attested that when an antioxidant is added to biodiesel, in that situation tert-Butylhydroquinone (TBHQ) and butylated hydroxytoluene (BHT), the PV increase has a linear correlation with time, which was measured in weeks of storage without air injection [33]. Likewise, the authors stated that biodiesel with a high PV has an even lower oxidative stability.

Pure biodiesel (B100) had 36 MJ/kg before the AOM assay; after it, the HHV of the samples was measured in the presence and absence of the extract. The oxidized biodiesel (B100*ox*) showed an HHV decrease of 14% compared to the original value of HHV of biodiesel (B100). The samples with antioxidant added did not show any significant changes. The results are listed in Table 4. On the other hand, the pure extract had an HHV of 23.6 MJ/kg. There is a small quantity of extract in biodiesel (1 g/L at the most); consequently, the possibility of reducing biodiesel HHV through the addition of extract is dismissed.

The loss of properties is a sign of biodiesel degradation. Partially-oxidized hydrocarbon chains reduce the biodiesel HHV, which is already lower than diesel HHV. Furthermore, loss of other properties such as viscosity and lubricity can lead to incorrect mechanical operation of engines [14].

B100 had a 2 h induction time at 110 °C, meaning that it did not meet the quality requirements, which is 3 h according to ASTM D 6741 and D 2274 standards, and 6 h [10,33] according to EN 14112. Nonetheless, this value was far exceeded by the samples treated with antioxidant, as seen in Table 4. These results are like those reported by van der Westhuizen, in which biodiesel from sunflower oil showed an OSI time of between 2 and 6 h [12]. The original low OSI of biodiesel can be attributed to the chemical composition, due to the high concentration of unsaturated FAME [7].

**Figure 6:**
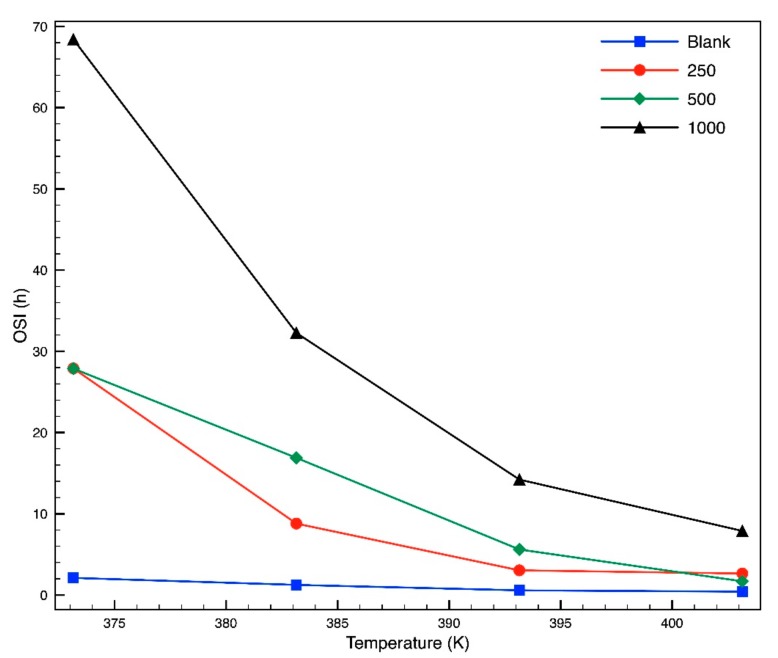
Oxidative stability index (OSI) at different temperatures and several different concentrations of *L. tridentata* extract.

**Figure 7:**
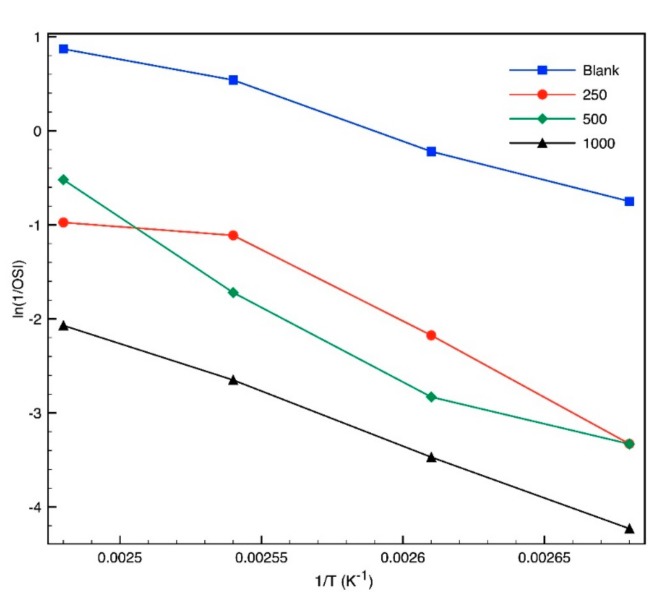
Linearized semilogarithmic inverse OSI vs. inverse absolute temperature equations.

OSI is reduced as the absolute temperature increases, given that rate constant (k) increases too, as it is indicated in Figure 6 and Figure 7. The OSI trend vs. temperature is exponential, according to the Arrhenius equation, which is indicated in Equation (2), while the ln (k) chart against the reciprocal of the absolute temperature approaches the line in a satisfactory level, according to Equation (3). The slope of this line is the relation between Ea and R. These parameters are shown in Table 5. Only 250 mg/L is needed to increase the activation energy more than 31% and Q_10_ by 26%. It proves that the creation of biodiesel degradation products is less sensitive to temperature and slower in the presence of the antioxidant. With a concentration of 500 mg/L or higher, the activation energy changed significantly.

Moreover, the biodiesel blended with 500 and 1000 mg/L of antioxidant behaved differently; thus OSI at 100 °C was not reached in continuous time of operation, even when the test lasted more than 24 h at 110 °C. An exponential regression equation was extrapolated by estimating three values, calculating an OSI time of 68 h at 100 °C. This data is higher than that reported by Karavalakis et al. [34], who documented OSI of 21 and 9 h at 110 °C in biodiesel with 1000 mg/L TBHQ and BHQ, respectively. Similar results were obtained by other authors, as is exhibited in Table 6. Botella et al. [35] reported that canola biodiesel raised its OSI up to 9.51 with the addition of 1000 mg/L of antioxidants. Roveda et al. [36] reported an OSI of soybean biodiesel of 3.7 h, meanwhile with the addition of 500 mg/L of butylated hydroxytoluene (BHT) or propyl gallate it increased. BHT was used in synergy with butylated hydroxyanisole (BHA) by Yang et al. [17] to increase the stability of raw oils. Mittelbach and Schober [37] used many commercial and synthetic antioxidants in canola biodiesel in a concentration of 1000 mg/L, increasing the OSI at 110 °C. The current authors report an OSI of 16.89 h with 500 mg/L of antioxidant and 32.27 h with 1000 mg/L at 110 °C.

The comparable values of 250 and 500 mg/L can be explained by considering that the optimal concentration has been overreached, considering that the 1000 mg/L sample produced a considerable amount of foam that distinguished it from the others, perhaps in consequence of the saturation of biodiesel with the extract. It became immiscible, which could have caused a negative result for its handling.

## 3. Materials and Methods

### 3.1. Extract Obtaining

#### 3.1.1. Plant Material Obtaining

Samples of a *L. tridentata* specimen were recollected from the aerial parts. The plant grows wildly in the Campeche Town, in Baja California, Mexico. It is a population located 14 km southern the border between California (USA) and Baja California (Mexico) on the parallel 32° N, longitude 115° W, in the Valley of Mexicali within the Sonoran Desert. The plant material consisted of leaves, branches, and flowers from the bush; these were rinsed with tap water to remove the dust and placed to dry in dark room at room temperature for 96 h.

#### 3.1.2. Maceration

The *L. tridentata* extract was obtained through cold maceration using methanol as a solvent in a 5:1 mass ratio for 4 h at 25 °C. Once this process was finished, the liquid was filtered through a Whatman Grade 4 filter paper at atmospheric pressure to be concentrated in a rotating evaporator afterward. Finally, the concentrated extract was placed on a stove at 70 °C to eliminate the solvent left. The extract was stored at 4 °C until its use.

### 3.2. Antioxidant Properties of the Extract

#### 3.2.1. Determination of Phenolic Acids by HPLC

A UFLC Shimadzu LC-20AD chromatograph (Shimadzu Scientific Instruments, Kyoto, Japan) equipped with a SIL-20AHT injector, a CNM-20A system controller, an SPD-M20A diode detector adjusted at 320 nm and an RP-18 Restek^®^ (4.6 × 250 mm ID, 5 µm) column (Restek Corporation, Bellefonte, PA, USA) were used to determine phenolic acids. The mobile phase was methanol and water with the addition of 0.1% of acids (pH = 2.78) and a flow rate of 0.6 mL/min. The results were interpreted through calibration curves to determine chlorogenic, caffeic, *p*-coumaric and ferulic acids. These were expressed in mg/g of extract. The test was performed with a single sample.

#### 3.2.2. Determination of the Free Radical Scavenging Capacity Through a DPPH Assay

The antioxidant capacity was determined through the free radical scavenging method according to Brand-Williams [38] including some adaptations. The assay involved the reaction of 0.01 mL of extract dissolution with 0.99 mL of 2,2-diphenyl-1-picrylhydrazyl (DPPH) dissolution for 30 min at room temperature in the dark; then the absorbance was measured at 517 nm using a Thermo Fisher Scientific^®^ Genesys 20 spectrophotometer (Thermo Scientific, Rochester, NY, USA). The decrease of absorbance in respect to a DPPH reference (without antioxidant added) is related to the radical-reducing capacity of the extract. The results were expressed as a percentage of inhibition, %Inh, as in Equation (1), where A_sample_ is DPPH absorbance after addition of an antioxidant, A_blank_ is the absorbance of blank solution from the sample and A_reference_ is the original absorbance of DPPH dissolution. Additionally, they are expressed in µmol of Trolox^®^ equivalent/g of dried extract (TEAC) through a calibration curve. The entire assay was performed with one blank solution and four replicate samples.

%Inh = (1 − (A_sample_ − A_blank_)/A_reference_). (1)

#### 3.2.3. Capacity of Ferric Reduction Through FRAP Assay

The antioxidant capacity was determined through the technique described by Benzie and Strain [39]. This technique has a principle about absorbance change caused by ion reduction from Fe^+3^ to Fe^+2^, which is present in the 2,4,6-tripyridyl-s-triazine-Fe(III) complex (TPTZ-Fe). The ferric reducing antioxidant power assay (FRAP) reagent was prepared with a 2,4,6-tris(2-pyridyl)-s-triazine (TPTZ) dilution in HCl 40 mM, to TPTZ concentration to reach 10 µM. Then, that volume of FeCl_3_ aqueous solution at 20 µM was added to it. Finally, a buffer solution of sodium acetate was added until a pH = 3.6 was reached. During the microplate assay, a sample of 15 µL of extract diluted with methanol, 15 µL of buffer solution and 270 µL of FRAP solution, along with a sample of blank solution were added. The reaction was performed for 30 min in the dark, later, the results were read at 590 nm on the Thermo Scientific Multiskan^®^ Spectrum (Thermo Scientific) plate reader. A higher absorbance means a higher reducing capacity. These results were expressed in µmol of Trolox^®^ equivalent/g of dried extract (TEAC) by means of a calibration curve. The entire assay was performed with four replicate samples.

#### 3.2.4. Capacity of Absorption of Oxygen Radicals Through ORAC Assay

The oxygen radical absorbance capacity (ORAC) assay was performed through the method described by Ou et al. [40], which consists in the measurement of 3 mL of fluorescence dilution containing the following: 21 µL from a 10 µM fluorescein dilution, 50 µL of 2,2′-azobis(2-amidinopropane) dihydrochloride (AAPH) at 600 mM and 30 µL of the extract diluted with methanol. All of them were gauged to 3 mL with a buffer solution 75 µM of phosphate ion. The fluorescence was measured with a Perkin Elmer LS45^®^ spectrofluorometer (PerkinElmer Inc., Waltham, MA, USA). The ORAC value was expressed as µmol of Trolox^®^ equivalent/g of extract (TEAC) through a calibration curve employing different Trolox^®^ concentrations.

#### 3.2.5. Total Phenols

A calibration curve of gallic acid was used to determine the total phenols, according to the technique proposed by Singleton and Rossi [41]. The following were deposited in a microplate: 15 µL of the extract diluted with methanol and later, 37 µL of Folin–Ciocalteu reagent and 128 µL of distilled water. After 5 min 120 µL of 7% *w*/*v* of Na_2_CO_3_ was added and reacted for 1 h in a dark room. The sample blanks were deposited in the same way. The absorbance was measured at 760 nm using a Thermo Scientific, Multiskan Spectrum^®^ spectrophotometer (Thermo Scientific). Finally, the phenol content was determined in mg of gallic acid equivalent per each gram of extract (GAE) through a calibration curve. The entire test was performed with four replicate samples.

### 3.3. Biodiesel Preparation and Characterization

#### 3.3.1. Biodiesel Preparation

Biodiesel (B100) was prepared with commercial canola oil as raw product through alkaline catalysis with sodium methoxide 0.5 M and an oil:methoxide volumetric ratio 5:1. This method was selected due to its economic and technical advantages compared to acid and enzymatic catalysis. The canola oil was bought in a local supermarket in Medellin, Colombia in March 2018 and was immediately processed. Its composition was determined through the gas chromatography-mass spectrophotometry (GC-MS) analysis of the FAME as is described further. Transesterification was performed for 1 h with an agitation of 400 rpm at 60 °C. Once the reaction was finished, glycerol was separated by decantation and biodiesel was washed with deionized water in a volumetric ratio 3:1 of water to biodiesel. Biodiesel was dried at 100 °C and stored.

#### 3.3.2. GC-MS

An Agilent 6890N^®^ gas chromatographer (Agilent Technologies Inc., Wilmington, USA) was used to characterize the FAME components of biodiesel, attached to it was a 5973N^®^ selective mass detector (Agilent Technologies Inc., Wilmington, NC, USA). This procedure was based on ASTM E 2997 [42]. Helium was used as carrier gas with an automatic splitless injection system and a 30-m long DB-1MS^®^ column whose inner diameter measured 0.25 mm and had a film thickness of 0.25 µm. A 3 µL volume was injected. The temperature of the injector and detector was 250 and 300 °C, respectively. The oven temperature was settled at 70 °C for 2 min, and after that, it was heated on a temperature slope from 8 °C/min to 300 °C. The oven remained at 300 °C for 30 min. The chromatogram peaks were identified by means of the times of retention and atomic spectrum.

### 3.4. Oxidative Stability of Biodiesel

Dilutions of the *L. tridentata* extract in biodiesel were prepared in concentrations of 250, 500 and 1000 mg/L. Additionally, a biodiesel sample without extract (B100) was set as blank. Samples of 30 mL were taken from each dissolution, and its oxidative stability was measured through the active oxygen method (AOM) with an aeration flow of 1150 mL/min at 100 ± 1 °C. The oxidation progress was monitored through peroxide value (PV) and higher heating value (HHV).

#### 3.4.1. Peroxide Value (PV)

The analysis technique described by Shantha and Decker [43] was used for the peroxide value (PV) monitoring. An amount of 0.3 g of oil was taken from each of the oil samples; these were agitated in a vortex until diluted in 3.5 mL of a mix consisting of seven parts chloroform to three parts methanol. A 500 µL aliquot was taken from the resulting blend and added 25 µL of a dissolution containing 0.144 M of FeSO_4_-7H_2_O and BaCl_2_ in HCl 0.4 M and 25 µL of NH_4_SCN 0.44 M. This solution was agitated afterward and allowed to stand for 10 min in the dark. The absorbance was measured at 500 nm with a Thermo Scientific, Multiskan Spectrum^®^ spectrophotometer (Thermo Scientific) and was compared to a calibration curve which had known concentrations of Fe^+3^. A higher absorbance means a higher presence of Fe^+3^, therefore, a higher PV. Additionally, the change of color to red is evidence of a high PV. Peroxide value was expressed as milliequivalents of O_2_/kg of sample (meO_2_/kg). The entire assay was performed by triplicate.

#### 3.4.2. Higher Heating Value

The biodiesel higher heating value (HHV) was measured before and after the AOM assay for the blank and the samples with antioxidant. For this, the isoperibolic method was performed using an IKA-Werke C2000 Basic^®^ calorimeter (IKA-Werke, Wilmington, DE, USA) at 25 °C with 3 MPa oxygen pressure. The pure extract higher heating value was also determined. The entire assay was performed by triplicate.

#### 3.4.3. Oxidative Stability Index

The oxidative stability index (OSI) was determined using a Metrohm Rancimat 679^®^ (Metrohm, Herisau, Switzerland). Four samples of 3 g of biodiesel were used: three samples with a concentration of 250, 500 and 1000 mg/L of antioxidant and a blank solution, and each sample was deposited in the reaction vials. These underwent a bubbling airflow of 10 L/h. The exit stream dragging the volatile material was transported into tubes with deionized water, where the conductivity was monitored. The OSI was defined as the time in which FAME degradation products increase water conductivity, also called induction time. The assay was carried out at 100, 110, 120 and 130 °C.

##### Effect of Temperature on OSI

According to the Arrhenius equation, the formation of FAME degradation products is related to the inverse of the temperature, as shown in Equation (2):

1/OSI = A × exp((−Ea)/RT),(2)

where OSI is the induction time in h, A is the Arrhenius constant, T is the absolute temperature in K, Ea is the activation energy, and R is the universal gas constant (8.314 J/mol K).

The Equations (3) and (4) proposed by Heidarpour et al. [44,45] establish that the natural logarithm of induction time has a linear correlation with temperature, as well as the reciprocal of the natural logarithm of induction time has with the reciprocal of temperature, according to the equations:

ln (OSI) = aT + b,(3)

ln k = ln(1/OSI) = ln (A) − Ea/(RT).(4)

If Equation (4) is written in a straight-line form, where the slope is m = Ea/R and whose independent variable is reciprocal of temperature, it results in the Equation (5):

ln (k) = ln (A) − m(1/T).(5)

The inverse of OSI is the rate constant, specifically of the biodiesel oxidation products which are responsible for conductivity increase, that is k = 1/OSI. The speed of formation of these compounds is sensitive to temperature [46]. The effect of that temperature is determined by the Q_10_ parameter, which in turn, can be defined by Symoniuk et al. [45] in Equation (6):

Q_10_ = (k_2_/k_1_)^^(10/(T^_2_^− T^_1_^)^. (6)

The values for k are measured at two temperatures, T_2_ and T_1,_ therefore, T_2_ > T_1_.

## 4. Conclusions

A vegetable extract was obtained from *L. tridentata* by maceration using methanol. The results of the DPPH, FRAP and ORAC methods showed that the extract performs as an antioxidant in biodiesel. The antioxidant capacity through the ORAC method was higher than that obtained by the DPPH method. A probable cause of this is the oversize of many molecules located in extracts, such as phenolic acids, terpenes, and flavonoids, that does not allow them to scavenge small radicals like DPPH. The FAME of biodiesel were identified and quantified through a GC-MS, and they are suitable to perform oxidative stability assays because the unsaturated FAME are more prone to oxidation than saturated ones due to its chemical composition. On the AOM assay, the PV of biodiesel samples with 250 mg/L of antioxidant remained low after 20 h and extended 3.79 times the necessary time to reach a PV of 100 meO_2_/kg. It indicates that biodiesel did not start the exponential phase of peroxide generation with the antioxidant addition. Biodiesel OSI was measured, and although it did not accomplish the standard minimum, it was possible to overreach the requirements of the standards due to the addition of the antioxidant. The HHV was not affected for AOM assay conditions in the samples with antioxidant added. Despite in this work, the antioxidant was assessed only on canola oil biodiesel, it is expected a similar effect on the oxidative stability of biodiesel from other sources with a high concentration of unsaturated FAME. The most outstanding feature of this research is the fact that *L. tridentata* extract is a natural and green alternative to synthetic antioxidants such as TBHQ and BHA. This extract is a useful, relatively abundant and renewable resource. The use of the methanolic *L. tridentata* extract in biodiesel improves its low oxidative stability and extends its storage shelf life.

## Figures and Tables

**Table 1:** Antioxidant activity of methanol extract of *L. tridentata*.

Method	Result
DPPH	10.14 ± 0.1 TEAC/g
FRAP	172.1 ± 0.13 TEAC/g
ORAC	50,770 ± 4.2 TEAC/g
Total Phenols	211.18 ± 0.39 GAE/g

**Table 2:** Phenolic compounds identified by high-performance liquid chromatography (HPLC) extract on the methanolic extract of *L. tridentata*.

Compound	Concentration (mg/g)
**Chlorogenic Acid**	0.920
**Caffeic Acid**	2.288
***p*-Coumaric Acid**	0.916
**Ferulic Acid**	0.849
**Catechin**	1.967
**Epicatechin**	1.866
Total	8.806

**Table 3:** Biodiesel composition by gas chromatography-mass spectrophotometry (GC-MS).

RT (min)	Methyl Ester Name	Relative Abundance (%)
32.24	9-Octadecenoic acid (z) methyl ester	46.35
32.14	9,12-Octadecadienoic acid, methyl ester	23.54
30.24	Hexadecanoic acid, methyl ester	9.04
32.49	Octadecanoic acid, methyl ester	5.46
34.08	11-Eicosenoic acid, methyl ester	3.12
Others		4.20

**Table 4:** Higher heating value of biodiesel before and after active oxygen method.

	B100	B100o*x*	Biodiesel 250 mg/L	Biodiesel 500 mg/L	Biodiesel 1000 mg/L
Average (MJ/kg)	36.11	31.17	35.94	35.59	36.48
SD	0.08	1.35	0.14	0.19	0.25

**Table 5:** Thermodynamic parameters of oxidative stability index.

Sample Concentration (mg/L)	T (°C)	OSI (h)	Linear Equation			Ea (kJ/mol)	Q_10_
			m	ln A	R^2^		
Blank	100	2.11	8457	−21.90	0.98	70.32	1.76
	110	1.25					
	120	0.58					
	130	0.42					
250	100	27.90	12,296	−29.81	0.93	102.23	2.38
	110	8.80					
	120	3.04					
	130	2.65					
500	100	27.88	14,249	−34.64	0.96	118.47	2.65
	110	16.89					
	120	5.61					
	130	1.69					
1000	100	68.41 *	10,989	−25.23	0.99	91.37	2.08
	110	32.27					
	120	14.22					
	130	7.89					

* extrapolated value.

**Table 6:** Comparison between different antioxidants for oils and biodiesel.

Material	Antioxidant	Concentration (mg/L)	Blank OSI (h)	OSI with Antioxidant (h)	Reference
Canola biodiesel	4-allyl-2,6-dimethoxyphenol	1000	5.2	7.45	Botella et al. [35]
	Catechol	1000	5.2	9.51	
Soybean biodiesel	BHT	500	3.7	7	Roveda et al. [36]
	Propyl gallate	500	3.7	10	
Soybean oil	Rosemary extract	400	2.2	3.40	Yang et al. [17]
	BHA+BHT	400	2.2	2.90	
Canola biodiesel	THBQ	1000	9.15	38.53	Mittelbach et al. [37]
	Propyl gallate	1000	9.15	27.36	
	BHA	1000	9.15	24.30	
Canola biodiesel	*L. tridentata* methanolic extract	1000	1.25	32.27	Current authors
		500	1.25	16.89	
